# A Cylindrical, Inner Volume Selecting 2D-T_2_-Prep Improves GRAPPA-Accelerated Image Quality in MRA of the Right Coronary Artery

**DOI:** 10.1371/journal.pone.0163618

**Published:** 2016-10-13

**Authors:** Andrew J. Coristine, Jerome Yerly, Matthias Stuber

**Affiliations:** 1 Department of Radiology, University Hospital (CHUV) / University of Lausanne (UNIL), Lausanne, VD, Switzerland; 2 CardioVascular Magnetic Resonance (CVMR) research centre, Centre for BioMedical Imaging (CIBM), Lausanne, VD, Switzerland; Generalitat Valenciana, SPAIN

## Abstract

**Background:**

Two-dimensional (2D) spatially selective radiofrequency (RF) pulses may be used to excite restricted volumes. By incorporating a "pencil beam" 2D pulse into a T_2_-Prep, one may create a "2D-T_2_-Prep" that combines T_2_-weighting with an intrinsic outer volume suppression. This may particularly benefit parallel imaging techniques, where artefacts typically originate from residual foldover signal. By suppressing foldover signal with a 2D-T_2_-Prep, image quality may therefore improve. We present numerical simulations, phantom and *in vivo* validations to address this hypothesis.

**Methods:**

A 2D-T_2_-Prep and a conventional T_2_-Prep were used with GRAPPA-accelerated MRI (R = 1.6). The techniques were first compared in numerical phantoms, where per pixel maps of SNR (SNR_multi_), noise, and g-factor were predicted for idealized sequences. Physical phantoms, with compartments doped to mimic blood, myocardium, fat, and coronary vasculature, were scanned with both T_2_-Preparation techniques to determine the actual SNR_multi_ and vessel sharpness. For *in vivo* experiments, the right coronary artery (RCA) was imaged in 10 healthy adults, using accelerations of R = 1,3, and 6, and vessel sharpness was measured for each.

**Results:**

In both simulations and phantom experiments, the 2D-T_2_-Prep improved SNR relative to the conventional T_2_-Prep, by an amount that depended on both the acceleration factor and the degree of outer volume suppression. For *in vivo* images of the RCA, vessel sharpness improved most at higher acceleration factors, demonstrating that the 2D-T_2_-Prep especially benefits accelerated coronary MRA.

**Conclusion:**

Suppressing outer volume signal with a 2D-T_2_-Prep improves image quality particularly well in GRAPPA-accelerated acquisitions in simulations, phantoms, and volunteers, demonstrating that it should be considered when performing accelerated coronary MRA.

## Introduction

In recent years, cardiac magnetic resonance imaging has benefitted greatly from the adoption of rapid imaging strategies, such as SENSE [1] and GRAPPA [2]. In the fundamental trade-off between signal-to-noise ratio (SNR), spatial resolution, and acquisition time, parallel imaging allows one to acquire images more quickly at the cost of decreased SNR. This is achieved by regularly undersampling k-space, such that aliased, reduced field-of-view (rFoV) images are obtained. By exploiting the different sensitivities of individual coils in the receiver array, the aliased image can then be "unfolded", such that the original image may be reconstructed [3]. In SENSE, this is done directly from the aliased images, whereas in GRAPPA, this is done in k-space. Both techniques lead to a reduction in the overall SNR that is proportional to the square root of the acceleration factor, R. However, there may also be an additional, nonuniform decrease in SNR that depends upon the object structure and the coil-geometry. Following the approach of Breuer *et al*. [4], this additional SNR loss, described by the GRAPPA g-factor, can be defined as follows:

$$g=\frac{SNR_{full}}{SNR_{acc}\sqrt{R}}$$ [1]

and may originate from residual foldover artefacts after GRAPPA reconstruction. Reducing the signal intensity of aliased structures may therefore reduce the g-factor of accelerated images, and in turn, improve image quality.

One potential approach to reducing these aliasing artefacts might be to spoil signal from outside of the region of interest. This could possibly be achieved with outer volume suppression [5,6,7], or by restricting the excitation volume, such as with two-dimensional spatially selective RF pulses ("2D pulses") [8,9,10]. Two dimensional pulses have already been proposed for a variety of applications, including cardiovascular uses like respiratory navigator gating [11,12,13], virtual dye angiography [14], cardiac [15,16] and vessel wall imaging [17,18,19,20,21], and for spin labelling [22,23,24,25,26]. They have also been shown to improve image quality by reducing signal from nearby anatomical structures [16], which may be sources of unwanted artefacts (e.g. respiratory ghosting). A significant limitation of 2D pulses, however, is their lengthy duration. If they are used as RF excitation pulses, they inevitably result in lengthy repetition times (TR), and therefore, a longer total scan time. This counteracts the very benefits that they might otherwise provide. However, a workaround has recently proposed [27] that incorporates a 2D pulse into a T_2_-preparation module (or T_2_-Prep) [28]. This approach, dubbed the "2D-T2-Prep", permits outer volume suppression (OVS) while preserving the contrast of the T_2_-Prep.

Although the 2D-T_2_-Prep has previously been shown to improve image quality in non-accelerated imaging (e.g. through a reduction in respiratory ghosting) [27], its outer volume suppression (OVS) may be of even greater use in parallel imaging. Reducing the signal intensity of aliased structures may therefore reduce the g-factor of GRAPPA-accelerated images, and in turn, improve image quality. While this idea was postulated in [27], it has yet to actually be quantitatively explored.

We therefore hypothesize that the 2D-T2-Prep will improve GRAPPA-accelerated image quality when compared to a conventional T_2_-Prep, even more than in the non-accelerated case. This hypothesis was tested in numerical simulations, phantom validations, and *in vivo* MRA of the right coronary artery (RCA).

## Materials and Methods

Permission from the Institutional Review Board, namely the "Commission cantonale d'éthique de la recherche sur l'être humain" (www.cer-vd.ch), was obtained for all *in vivo* scans, and written informed consent was obtained from all volunteers prior to the procedure.

### Background

**Fig 1:**
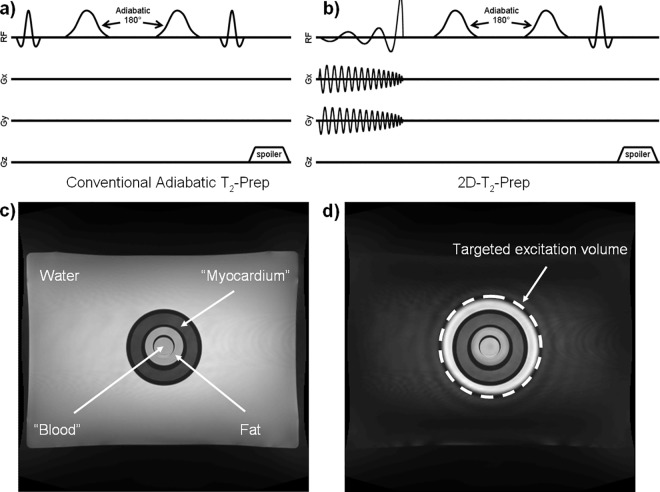
**a)** Pulse sequence diagrams for the conventional adiabatic T_2_-Prep and **b)** the 2D-T_2_-Prep. In the 2D-T_2_-Prep, the initial tip-down pulse has been replaced by a spatially selective jinc pulse, which excites a cylindrical volume (12.0 cm). The final pulse, which remains non-selective, restores this T_2_-Prepared cylinder while tipping outer volume magnetization into the transverse plane, where it is spoiled. Both of the effective T_2_-Prep durations remain the same and either may be used prior imaging. **c,d)** A 50 scan average of both techniques. In these images, a homebuilt phantom, with compartments doped to mimic blood, fat, and myocardium, was placed in a large water bath and imaged with a 2D sequence preceded by either the **c)** conventional T_2_-Prep or the **d)** 2D-T_2_-Prep.

When performing coronary MRA, a conventional imaging sequence may consist of a navigator for respiratory motion suppression, an ECG-triggered segmented k-space signal readout, and a T_2_-preparation module to enhance contrast between blood and myocardium [29,30,31,32,33]. An adiabatic T_2_-Preparation technique [34], consisting of a 90° excitation pulse, followed by two hyperbolic secant adiabatic 180° pulses [35], and a -90° restoration pulse (Fig 1A), has previously been shown to be quite effective [36,37,38,39]. This "conventional" adiabatic T_2_-Prep adds T_2_-weighting, enhancing contrast between the blood and myocardium without the need for a long echo time (TE) (Fig 1B). In turn, this allows for a shorter TR and a more rapid data acquisition. The 2D-T_2_-Prep [27] functions similarly, except that a 2D pulse is used as the initial excitation pulse.

Specifically, the first RF pulse of the conventional adiabatic T_2_-Prep is replaced with a jinc pulse and spiral gradients (Fig 1C). This excites a cylindrical volume. Meanwhile, the final RF pulse remains non-selective. As a result, it restores the T_2_-Prepared cylinder, while also tipping the (formerly longitudinal) magnetization outside of the cylinder into the transverse plane. A spoiler then destroys that transverse magnetization. Consequently, at the end of the 2D-T_2_-Prep, only the initial cylinder of T_2_-prepared longitudinal magnetization remains (Fig 1D). Outer volume signal has thus been suppressed prior to imaging at no additional temporal cost. Additionally, to partially compensate for T_1_ signal recovery, the excitation and restoration pulses may be increased beyond 90°; as per (27), ±100° pulses were used in this study.

In this study, the 2D-T_2_-Prep, and its conventional counterpart, were used prior to normal and GRAPPA-accelerated MRI, in numerical simulations, phantom studies, and in healthy adult volunteers.

### Numerical Simulations

A parallel imaging undersampling and reconstruction simulation was first designed using a commercial software package (MATLAB 7.11, The MathWorks Inc., Natick, MA, United States). This simulation was used to predict the results of acquiring GRAPPA-accelerated images of a numerical phantom, for acceleration factors of R = 1,2,3,4,5, and 6, for idealized versions of both the conventional and the 2D-T_2_-Prep (i.e. to determine the theoretical limit). The numerical phantom (Fig 1), based on a 50 scan average of a real phantom (see below), contained regions that mimic the MRA properties of blood, myocardium, and fat. That 50-scan average was treated as the gold standard reference image for the conventional T_2_-Prep. For each simulation, a different set of Gaussian coil noise was added to k-space, evenly distributed throughout a 16 channel coil array, such that the final image would have a mean SNR of 50 [40,41,42]. The simulated 2D-T_2_-Prep itself consisted of a uniform cylindrical excitation of radius 6.0 cm, with perfect background suppression, with coil noise added to k-space as per the conventional T_2_-Prep. GRAPPA acceleration simulations were run 500 times for each T_2_-Prep technique, at every acceleration factor (2×500×6 = 6000 simulations), to generate a large set of images. By analyzing these repeated simulations, per pixel maps of SNR (i.e. SNR_multi_), noise, and g-factor were predicted by calculating the standard deviation across each pixel after image reconstruction. Specifically, the pixel noise was defined as the standard deviation of a given pixel's signal intensity across all 500 simulations. From there, the SNR_multi_ was determined and the g-factor calculated using Eq 1.

For these simulations, the accelerated images were created by performing a Cartesian undersampling of the fully sampled k-space data along the phase-encoded axial direction. To synthesize the missing k-space values, the GRAPPA calculations applied a linear combination of acquired neighbouring k-space data from the coils using the formalism introduced by Lustig *et al*. [43], where *β* = 0.01 was used as the Tikhonov regularization term. To determine the GRAPPA reconstruction weights, a region of 24×24 pixels at the origin of k-space was always fully sampled (25×25 for R = 5). The size of the calibration kernel was selected so as to match those used in the actual phantom experiments, as described below.

After the simulations had been run, ROIs covering each "tissue" (blood, myocardium, and fat) were automatically segmented. Within these ROIs, the SNR_multi_ of each pixel was calculated, such that the mean SNR_multi_ of each tissue region could be estimated. The percent difference in the mean SNR_multi_, between the conventional and the 2D-T_2_-Prep, was next calculated and plotted versus the GRAPPA acceleration factor. A paired 2-tailed Student’s t-test was used to analyze the percent differences, with a P <0.05 considered statistically significant. These simulations were repeated for a 2D-T_2_-Prep radius of 12.0 cm (hereinafter referred to as the "double radius" 2D-T2-Prep), and the SNR_multi_, noise, and g-factor maps were displayed alongside the above simulations for visual comparison.

### Phantom Study

Two sets of phantom experiments were performed. The first set of experiments was used to compare SNR_multi_ between the two T_2_-Prep techniques, as was done in the numerical simulations above. A second set of experiments was used to compare vessel sharpness, as per the *in vivo* experiments below.

For the first set of phantom experiments, a home-built cardiac phantom was constructed, with concentric compartments doped to mimic blood, myocardium, and fat. This phantom was ~10 cm in diameter, with outer and middle Plexiglas borders of 0.5 cm each, surrounding the 1.5 cm thick myocardium. The fat section was approximately 1 cm wide, separated from the 2.7 cm diameter blood section by a 2 mm plastic border. Relaxation times, which were previously measured using inversion recovery and spin echo sequences, were as follows: Myocardium: T1 = 1127±11 ms, T2 = 35.5±0.7 ms; Fat: T1 = 213±5 ms, T2 = 45.8±1.2 ms; Blood: T1 = 1469±24 ms, T2 = 246±7 ms. Both the myocardium and blood sections were constructed from agar gel doped with nickel chloride, as per Kraft *et al* [44], with sodium azide added to prevent bacterial growth (all from Sigma Aldrich, St. Louis, MO). This corresponded to a pre-boiling concentration of 7.5 mg NiCl2 / 100 mL with 0.5% agar w/v for blood, and 9.5 mg NiCl2 / 100 mL with 4.5% agar w/v for myocardium. Fat was modeled using baby oil (Johnson and Johnson, New Brunswick, NJ).

This cardiac phantom was placed in a large rectangular water bath, as shown in Fig 1C. It was scanned 50 times at GRAPPA acceleration factors of 1,2,3,4,5, and 6, for both the conventional T_2_-Prep and the 2D-T2-Prep (6.0 cm radius). The 50-scan average of the non-accelerated (R = 1) conventional T_2_-Prep was also used as the gold standard image for the numerical simulations above. All physical experiments (50x6x2 = 600 total scans) were performed on a 1.5 T clinical scanner (MAGNETOM Aera, Siemens AG, Healthcare Sector, Erlangen, Germany) using an artificial ECG signal (60 bpm) for triggering, a 2D gradient echo imaging sequence, 18 channel anterior chest coil + 12 element spine coil, FoV 384x384 (matrix 384x384), 4.0 mm slices, TE T_2_-Prep = 40 ms, RF excitation angle 20°, and TE/TR/T_acq_ = 3.4/8.7/69 ms.

When scanning, the 2D pulse parameters were chosen to mimic the dimensions of an adult heart and torso: a cylinder radius of 6.0 cm, aliasing rings of radius 24.0 cm, and a jinc-shaped RF pulse with 5 zero-crossings to ensure a consistent and rectangular excitation. These values corresponded to a uniform density spiral trajectory with 15.6 turns and a 5.8 ms RF pulse duration. All other imaging parameters were as per the *in vivo* study described below, with a simulated RR interval of 1000 ms.

Once 50 images had been acquired for each T_2_-Prep/acceleration factor combination, it was possible to calculate per pixel maps of SNR (i.e. SNR_multi_), noise, and g-factor, by determining the standard deviation across each pixel. For each "tissue" in the phantom, an ROI was chosen and the mean SNR_multi_ within that region was calculated. As with the numerical simulations, the percent difference between the conventional and the 2D-T_2_-Prep was calculated and plotted, and A paired 2-tailed Student’s t-test was used to compare the means, with a P <0.05 considered statistically significant.

The second phantom study was performed to study the effects of the 2D-T_2_-Prep on vessel sharpness. For this study, the imaging parameters were as in the above experiments, though a segmented, volume-targeted 3D gradient echo imaging sequence was used [33], with 1.5 mm reconstructed slices (3.0 mm acquired), 24 mm volume thickness, water-selective RF excitation pulses of 20°, and TE/TR/T_acq_ = 5.2/11.6/93.0 ms. The sequence was triggered to an artificial ECG signal with a cardiac frequency of 60 beats/minute, so as to accurately represent a cardiac imaging sequence. A variety of objects were added to the imaging volume, so as to represent a more complex anatomical structure (see below). Notably, a small length of tubing, with a ~6 mm inner diameter, was added and filled with a nickel sulphate solution (3.75 g NiSO_4_ + 5 g NaCl per 1000 g H_2_O) so as to produce a bright "coronary-like" structure. It was placed such that the vessel curved within the imaging plane, with portions running perpendicular to either the frequency or phase encode directions. The phantom was then imaged at acceleration factors of R = 1,2,3,4,5, and 6, using both the conventional and the 2D-T_2_-Prep, and each scan was repeated 10 times. All images underwent a surface reformat so as to fit the "vessel" to a single plane for analysis with the Soap-Bubble quantitative software tool [45] to determine the vessel sharpness (VS). This was measured along 3 cm portions of the vessel, which were either perpendicular to the frequency axis or the phase (i.e. accelerated) axis. The two T_2_-Prep techniques were compared by calculating the absolute and relative % differences in VS between each technique, along each axis, for the 10 scans. A paired 2-tailed Student’s t-test was used to compare the two techniques, with a P <0.05 considered statistically significant. Afterwards, the % differences in VS between the two T_2_-Prep techniques, at each acceleration factor, and for each vessel orientation, were then plotted.

### *In Vivo* Study

For *in vivo* experiments, the RCA was imaged in 10 healthy adult subjects (ages 19–32, 6 female). Permission from the Institutional Review Board was obtained for all *in vivo* scans, and written informed consent was obtained from all volunteers prior to the procedure. Imaging parameters were as in the phantom vessel sharpness experiments, though only acceleration factors of R = 1,3, and 6 were compared due to time constraints. Additionally, the experiments were both respiratory- and ECG-gated, with an end-expiratory navigator gating acceptance window of ±2 mm selected to minimize respiratory motion artefacts, using a slice tracking factor of 0.60 [46]. The scan times varied for each volunteer, depending on heart rate and respiratory pattern (i.e. end-expiratory percentage), with each acquisition taking 384 heartbeats to acquire for an R = 1 [(8 segments/heartbeat)^-1^ * 384 segments/z-partition * 8 z-partitions/acquisition]. Thus, for a heart rate of 72 BPM and a respiratory acceptance of 40%, one can calculate the base scan duration to be 13min20s [384 heartbeats * (72 heartbeats/min)^-1^ * (0.4)^-1^], 4min27s for R = 3, and 2min13s for R = 6. Similar to the phantom vessel sharpness experiments, all images were reformatted and analyzed using the Soap-Bubble quantitative software tool [45]. For each volunteer, a continuous section of the coronary was identified, both visually and with Sopabubble, where VS could be evaluated across that section using either T_2_-Prep technique, and the % differences in VS was calculated for each acceleration factor. A paired 2-tailed Student’s t-test was used to compare these VS differences, with a P < 0.05 considered statistically significant.

## Results

### Numerical Simulations

**Fig 2:**
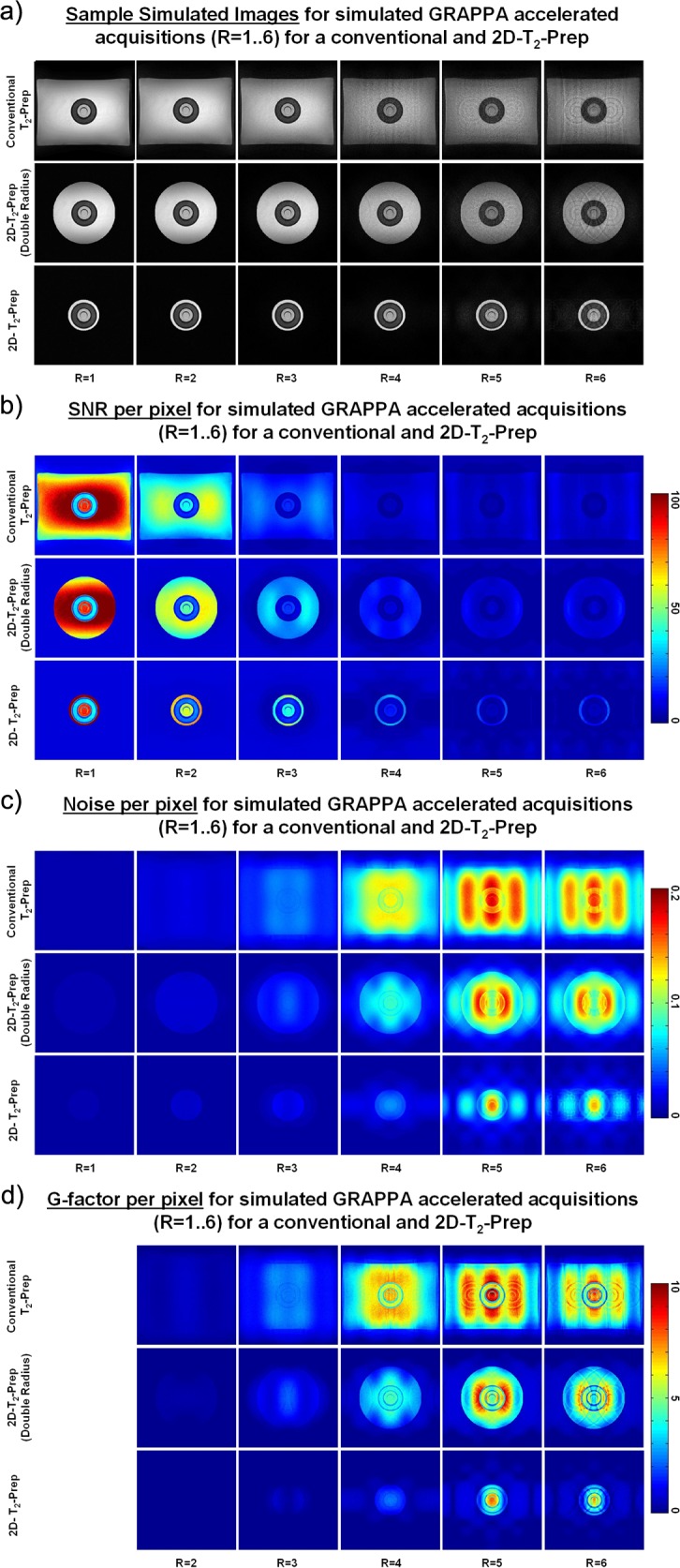
Numerically simulated GRAPPA-accelerated acquisitions of a phantom. Random coil noise was added to the 50 scan average described in Fig 1C) for the conventional T_2_-Prep as well as for 2D-T_2_-Prep modules of radius 6.0 and 12.0 cm. GRAPPA-accelerated image acquisitions were simulated for acceleration factors of R = 1 to 6. Sample images can be seen in **a)**, with corresponding per-pixel maps of **b)** SNR, **c)** Noise, and **d)** G-factor. Note that accelerated image quality appears to improve as the excitation volume decreases, though the position and nature of GRAPPA artefacts also appear to shift.

In simulations, the 2D-T_2_-Prep significantly improved image quality for accelerated acquisitions as compared to the conventional T_2_-Prep. Fig 2 illustrates this through comparative per-pixel maps of SNR, noise, and g-factor, for each acceleration factor. Through visual observation, it can be seen that the improvements in image quality appear to be correlated to the reduction in excitation volume, such that a greater outer volume suppression leads to higher image quality. That is, the standard (6.0 cm radius) 2D-T_2_-Prep improves image quality as compared to "Double Radius" 2D-T_2_-Prep, though both improve image quality as compared to the conventional T_2_-Prep. However, it can also be seen that the position and intensity of GRAPPA artefacts undergo spatial displacement when the excitation volume is changed, particularly in the case of the "Double Radius" 2D-T_2_-Prep. More specifically, when the imaged object changes (i.e. from a different OVS radius), the convolution with the aliasing kernel may lead to different aliasing intensities (even if the aliasing kernel stays the same).

**Fig 3:**
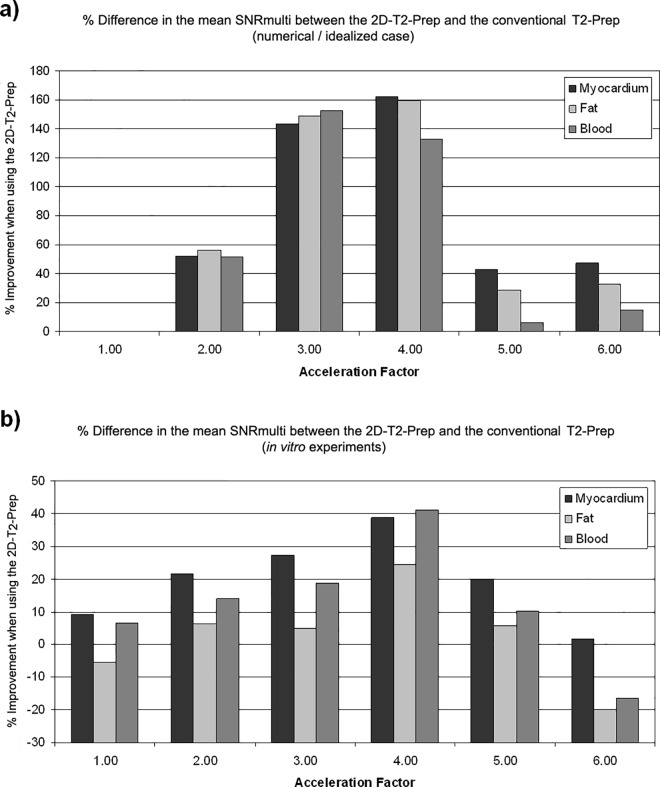
Improvement in the mean SNR_multi_, in regions of a tissue-mimicking phantom, when using a 2D-T_2_-Prep instead of a conventional T_2_-Prep, for GRAPPA accelerations of R = 1,2,3,4,5, and 6. Results are shown for both **a)** the simulated, ideal T_2_-Prep shown in Fig 2 and **b)** the corresponding physical phantom. Fig **a)** demonstrates the maximum possible signal improvement achievable, in the case of perfect background suppression with a 6.0 cm radius cylinder, whereas Fig **b)** demonstrates the actual results achieved in the physical phantom. In both cases the maximal SNR improvement appears to occur at R = 4, which also corresponds to the degree of outer volume suppression of the 2D-T_2_-Prep.

Nonetheless, for the conventional T_2_-Prep and the standard 2D-T_2_-Prep, the GRAPPA artefacts appeared to occur in similar locations. A quantitative numerical comparison was thus attempted between these two techniques. The SNR was first calculated on a pixel-by-pixel basis (SNR_multi_) and the SNR_multi_ within each tissue (blood, fat, and myocardium) was averaged for each T_2_-Prep. For example, at R = 2, this corresponded to a mean SNR_multi_ of 16.9 (conv.) vs. 25.7 (2D) for myocardium, 38.6 (conv.) vs. 60.3 (2D) for fat, and 39.9 (conv.) vs. 60.3 (2D) for blood. The % improvement in the mean SNR_multi_ (2D vs. conv.) was then plotted in Fig 3A. These % improvements were all statistically significant (P < 0.05) and by examining these improvements, it can be seen that the peak improvement occurs at acceleration factor of R = 4.

### Phantom Study

When the above comparison was repeated in phantom scans, there was also a maximal SNR_multi_ improvement at R = 4 (Fig 3B). Additionally, the change in the average SNR_multi_ per tissue was visually observed to follow a similar trend to that of the numerical simulations. All % differences in SNR_multi_, between the conventional and the 2D-T_2_-Prep, were found to be statistically significant (P<0.05).

**Fig 4:**
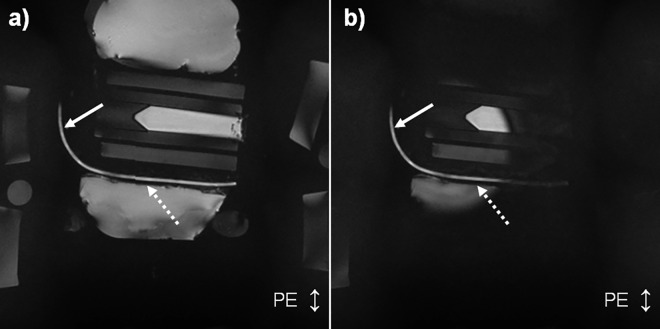
**Sample scan (R = 1) of the phantom used for vessel sharpness measurements, imaged using **a)** the conventional adiabatic T_2_-Prep and **b)** the 2D-T_2_-Prep.** Images were acquired with acceleration factors of R = 1,2,3,4,5, and 6, where the phase encoded (accelerated) direction is indicated by the two-headed arrow at the bottom right. Vessel sharpness was measured along the artificial vessel, in portions parallel to the accelerated direction (solid arrow) and perpendicular to the accelerated direction (dashed arrow).

**Fig 5:**
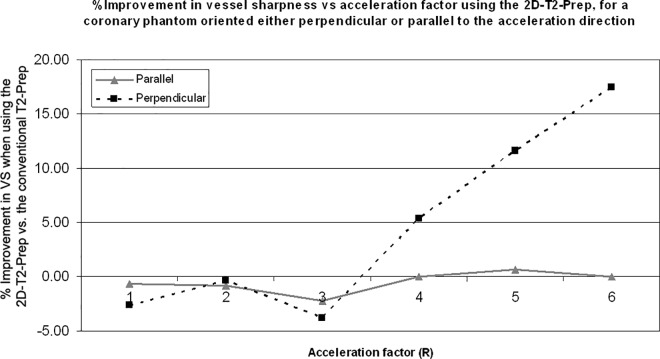
Improvement in the mean vessel sharpness, as measured in a coronary-mimicking phantom, when using a 2D-T_2_-Prep instead of a conventional T_2_-Prep, for GRAPPA accelerations of R = 1,2,3,4,5, and 6. Results are shown for a "vessel" section **a)** parallel to the accelerated direction (gray triangles) and **b)** perpendicular to the accelerated direction (black squares). As artefacts propagate along the accelerated direction, the parallel case acts as a vessel sharpness control, whereas the perpendicular case may be used to determine the relative benefit of the 2D-T_2_-Prep.

In the second set of phantom experiments (Fig 4), which measured vessel sharpness, it was found that for vessels oriented perpendicular to the accelerated direction, the higher the acceleration factor, the greater the relative increase in vessel sharpness from baseline when using a 2D-T_2_-Prep instead of a conventional T_2_-Prep (Fig 5, black squares). Conversely, for vessels running parallel to the accelerated direction (i.e. the control case), there was little change in the VS when comparing the two T_2_-Prep techniques (gray triangles).

### *In Vivo* Study

**Fig 6:**
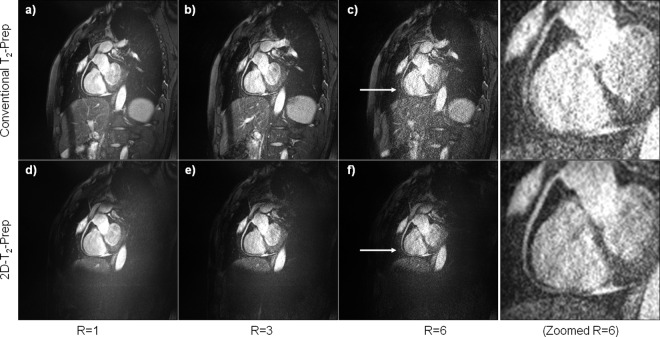
**Sample *in vivo* images of the RCA at various GRAPPA-acceleration factors, for both **a-c)** the conventional adiabatic T_2_-Prep and **d-f)** the 2D-T_2_-Prep.** The 2D-T_2_-Prep increased the mean vessel sharpness, as compared to the conventional T_2_-Prep, even in the non-accelerated case. However, this relative improvement was greater at R = 3 and greater still at R = 6, consistent with the phantom data shown in Fig 5. Note the significantly decreased conspicuity of the distal RCA, particularly for the R = 6 case, when using the conventional T_2_-Prep. Conversely, this is still quite visible with the 2D-T_2_-Prep (white arrows).

For *in vivo* images of the RCA (Fig 6), the mean vessel sharpness improved by 5% when using the 2D-T_2_-Prep rather than a conventional T_2_-Prep, for non-accelerated image acquisitions (Table 1). This result was consistent with those found in previous work [27]. However, the relative increase in vessel sharpness was even greater in accelerated image acquisitions. For an acceleration factor of 3, the mean vessel sharpness increased by 7% (P = 0.0005), and for an acceleration factor of 6, by 12% (P = 0.00002). Visually, these results can be appreciated in Fig 6, which shows sample images of the RCA acquired with both the conventional and the 2D-T_2_-Prep. While the image quality of each T_2_-Prep is comparable at an R = 1 (Fig 6A and 6D), the 2D-T_2_-Prep image shows an improved vessel delineation along the coronary boundary in the R = 3 case (Fig 6B and 6E). The improvement of the 2D-T_2_-Prep becomes even more evident at an acceleration factor of 6, where the distal portion of the RCA is quite noisy in the conventional T_2_-Prep and in fact, can scarcely be discerned (Fig 6C). However, the RCA can still be resolved quite well with the 2D-T_2_-Prep at an acceleration factor of 6 (Fig 6F). This trend of increasing vessel sharpness at higher acceleration factors was consistent with the phantom results described above.

**Table 1:** Improvement in RCA Vessel Sharpness using the 2D-T2-Prep for R = 1,3, and 6.

**Acceleration Factor**	1.00		3.00		6.00	
**T**_**2**_**-Prep version**	Conv.	2D	Conv.	2D	Conv.	2D
**Mean vessel sharpness %**	57.35	60.13	57.93	62.02	54.27	60.72
**Relative % difference in VS**	4.86		7.07		11.88	
**p-value**	0.0125		0.0005		0.00002	

Improvement in the mean vessel sharpness of the RCA in healthy volunteers, using either a conventional adiabatic T_2_-Prep or a 2D-T_2_-Prep, for GRAPPA acceleration factors of R = 1,3, and 6. Vessel sharpness improved for the 2D-T2-Prep, though by an even greater % for highest acceleration factors.

## Discussion

The combination of 2D-T_2_-Prep and GRAPPA improves image quality in accelerated coronary MRA. This has been demonstrated in numerical simulations, phantom studies, and *in vivo* experiments in human subjects. While the 2D-T2-Prep also improves image quality in non-accelerated imaging, it provides even greater improvements to GRAPPA-accelerated parallel imaging. Additionally, these benefits come at no extra time cost when used with T_2_ prepared cardiac imaging. Moreover, this principle—that outer volume suppression improves accelerated image quality—may potentially be of benefit to any form of accelerated image acquisition, in cases where a local ROI is targeted and information from adjacent tissues is either not required or detrimental to the overall image quality [47] in the region of interest.

The precise mechanism responsible for these improvements is hypothesized to be the reduction in excitation volume and the corresponding reduction in parallel imaging artefacts. While significant evidence has been provided to support this claim, unequivocally demonstrating this is challenging for several reasons. Parallel imaging artefacts may be displaced by a change in the structure of the object being imaged, the undersampling scheme adopted, and the acceleration factor used. A region of interest may therefore appear to improve in quality simply because a local artefact has moved elsewhere. Examples of this can be seen in Fig 2, where the GRAPPA "hotspots" appear in similar locations for both the conventional T_2_-Prep and the 6.0 cm radius 2D-T_2_-Prep, but are spatially displaced in the "Double Radius" 2D-T_2_-Prep. Likewise, in simulations, the image quality visually appears to improve when increasing the acceleration factor from 5 to 6. This fact—that parallel imaging aliasing may be moved [48]—has previously been exploited in CAIPIRINHA imaging [49] to shift local hotspots to regions outside the area of interest. In CAIPIRINHA imaging, the aliasing kernel is adapted in such a way that high-noise regions are displaced, whereas in the case of the 2D-T2-Prep, it may simply be that because the imaged object changes (i.e. from the different OVS radius), it is the *convolution* with the aliasing kernel leads to different aliasing intensities, even if the original aliasing kernel does not change. However, the net result is similar in each case; by shifting the positions and intensities of local hotpots, the 2D-T_2_-Prep has the potential to locally improve image quality in accelerated imaging.

However, the shifting of high g-factor regions may also be detrimental to image quality when they are shifted to regions of interest. This might also help to explain the slight decrease in vessel sharpness, at R = 3, in the second set of phantom experiments. Specifically, in Fig 5, starting at R = 1 and continuing to R = 6, there is a quasi-linear relationship between the improvement in VS and the acceleration factor for a vessel oriented perpendicular to the acceleration direction (dotted black line). However, at an R = 3, there is a sudden drop in the % VS improvement. A possible explanation is that a regional hotspot was moved onto the vessel at that particular acceleration factor. For reasons such as these, it may be challenging to perform regional comparisons across acceleration factors without changing other imaging parameters. While every effort has been made to address these concerns, they remain a fundamental and intrinsic limitation of this study.

This also highlights a shortcoming of the corresponding *in vivo* study. Specifically, it would have been interesting to see how image quality improves in different segments of the artery (e.g. proximal, medial, distal) with the new sequence, perhaps to see if orientation or proximity were affected. However, and particularly in the case of highly accelerated images, this was not always possible. In cases where a conventional T2-Prep was used, rather than the 2D-T2-Prep, image quality in parts of the vessel could be poor (as can be seen, for instance, in Fig 6C). This prevented Soapbubble from tracking and evaluating VS across the whole vessel. Instead, a continuous section of the coronary was identified, both visually and with Soapbubble, where VS could be evaluated across that same section, using either technique. An alternative may have been to use signal averaging, despite the apparent counter-intuitiveness of doing so with parallel imaging.

Another limitation of this study was the lack of SNR measurements *in vivo*. In simulations and phantom experiments, it was possible to perform repeated measurements to determine the true per pixel SNR (i.e. SNR_multi_). However, in volunteers, measuring SNR_multi_ was neither technically nor ethically justifiable, due to the repeated scan requirements. Meanwhile, conventional SNR measurements (signal/σ_background_) are not technically valid, due to the spatial dependence of background noise in parallel imaging. Instead, vessel sharpness was chosen as a surrogate measure of image quality, and an effort was made to compare this to SNR improvements in phantoms by examining the VS in a home-built mock coronary phantom. As compared to the conventional T_2_-Prep, the 2D-T_2_-Prep improved the relative VS by the greatest amount at the highest acceleration factors. SNR, meanwhile, appeared to improve most when the acceleration factor corresponded to the reduction in excitation volume. For instance, in Fig 3, it can be seen that the % improvement in SNR, of the 2D-T2-Prep vs. the conventional T2-Prep, occurs at an acceleration factor of R = 4. This also corresponded to the degree of outer volume suppression (OVS) of the 2D-T_2_-Prep. In other words, the ratio of excited tissue area to suppressed tissue area, along the phase-encoding axis, is also approximately 4. This apparent contradiction may nonetheless be accurate. VS is most adversely affected by blurring or an increase in background signal (e.g. residual foldover), as both of these obscure the transition from the lumen blood pool to the surrounding tissue. Conversely, the apparent SNR of a tissue may benefit from blurring as it may "smooth out" local signal fluctuations. Likewise, the apparent SNR may increase if there is an increase in background signal (e.g. from signal foldover). This may explain why SNR and VS are not necessarily correlated.

In this work, the background suppression of the 2D-T_2_-Prep, while effective, was incomplete. This may be due to B1 inhomogeneities and/or T_1_ signal recovery after the 2D-T_2_-Prep [27]. It may also be because 2D selective excitation pulses are not perfect in practice. As such, the current implementation may be less effective than theoretically possible. In numerical simulations, where the background suppression and excitation uniformity were assumed to be perfect, the SNR_multi_ improvement of the 2D-T_2_-Prep (vs. the conventional T_2_-Prep) was much greater than in phantom studies (Fig 3). This suggests that despite the significant results shown in this work, there is the potential for even greater improvement if the background suppression of the 2D-T_2_-Prep can be further improved. Conversely, it may also lead to unforeseen problems in situations where there is increased signal recovery or if B_1_ homogeneity worsens. For instance, in high BMI patients, fat signal recovery may become more problematic. Likewise, at higher field strengths (e.g. 3T), the greater B_1_ inhomogeneity may further impair complete background suppression.

Note that, the reduction of signal from outside the region of interest was achieved by incorporating a 2D excitation pulse into a 2D-T_2_-Prep. However, we did not study whether using a 2D RF pulse for image excitation would have produced similar results. Though the increase in scan time might currently preclude the clinical utility of 2D excitation in many approaches (e.g steady state free precession), 2D excitation pulses have nonetheless been previously used in spin-echo imaging [16] to good effect. It would be interesting to see if the results shown here—that the reduction in excited tissue volume particularly benefits parallel imaging—also hold true in the spin-echo case.

In fact, this hypothesis could be tested in any sequence with reduced excitation volumes, from simple inner volume selection techniques [7] to the outer volume suppression approach suggested by Smith et al [15]. The latter approach, which is similar in concept to a 0 ms 2D-T_2_-Prep, uses a non-selective adiabatic tip-down pulse, followed immediately by a 2D restoration, to attenuate signal outside a cylindrical region of interest. While Smith's OVS sequence was not aimed at T_2_-weighting, it nonetheless suppresses outer volume in a similar fashion. In fact, it might actually prove to be superior to the 2D-T_2_-Prep in cases where T_2_-weighting is unnecessary; the duration of the OVS magnetization preparation scheme is much shorter than that of the 2D-T_2_-Prep, as it consists solely of a tip-down/tip-up pulse train. In applications where T_2_ weighting is not necessary, Smith's OVS approach may more efficiently improve accelerated image acquisitions. However, this remains to be investigated in future studies.

An additional, perhaps more important limitation of this work was that it did not compare the 2D-T_2_-Prep to other signal saturation strategies. Specifically, a saturation band, consisting of a +90° slice selective excitation, followed by immediate signal spoiling, is often applied to the chest wall in cardiac imaging. In this study, the 2D-T_2_-Prep was compared to a conventional T_2_-Prep directly, whereas in a clinical setting, a conventional T_2_-Prep might also be accompanied by a saturation band. While saturation bands are generally only applied the anterior chest wall, or occasionally to both the anterior and posterior chest walls (such as in overweight patients), one could also imagine a situation where four saturation bands were applied, orthogonally around the heart, to achieve a similar effect to the 2D-T_2_-Prep. Nonetheless, it is worth considering that saturation bands require additional time, unlike the 2D-T_2_-Prep, which can be used as a direct substitution in place of a conventional T_2_-Prep. Saturation bands may also introduce other artefacts [50], require additional user time to place correctly, and may be less useful in radial imaging because of the repeated acquisition of the center of k-space. However, it may still be worth exploring other approaches to chest wall signal saturation.

It may also be interesting to consider a modified version of our 2D-T_2_-Prep, such as the one recently proposed by Luo et al [51]. That version uses a non-selective adiabatic BIR-4 tip-down pulse with a 2D restoration pulse (i.e. a "mirrored" version of our 2D-T_2_-Prep). That sequence benefits from a more homogenous excitation through the use of an adiabatic tip-down pulse, as well as from a shorter 2D pulse duration, the latter of which is achieved by exploiting a the conjugate symmetry described in [15]. This short 2D pulse duration may therefore make it more robust to signal loss during the 2D pulse in regions with very short T_2_* values (e.g. lung parenchyma). However, a limitation of Luo's variant is that tissues experience T_1_ recovery throughout the duration of the T_2_-Prep time. This may make it less suitable for longer T_2_-Prep durations, as background signal recovery will reduce the benefits gained from outer volume suppression.

Additionally, it may prove interesting to combine the 2D-T_2_-Prep with a different parallel imaging technique. In these studies, GRAPPA acceleration was used as a model for parallel imaging. In many ways, a SENSE acceleration would appear to be a more intuitive model, given that the 2D-T_2_-Prep directly suppresses signal within the image domain. However, this interesting combination remains to be investigated.

Another option would be to increase the dimensionality of the GRAPPA acceleration. In these experiments, images were only accelerated along the phase-encoding direction. However, as the dataset was collected as a 3D volume, the image could potentially be accelerated in two dimensions [52,53]. This would require changing the frequency encoding direction to be in the same direction as the 2D cylinder axis, so that both accelerated (phase-encoded) directions were perpendicular to the cylinder (i.e. AP and RL). Multidimensional acceleration has previously been applied to cardiovascular parallel imaging [54,55,56] and thus might be interesting to investigate in combination with the 2D-T2-Prep.

Notably, it is also possible that suppressing undesirable dynamic data from outside of the target ROI might lead to reduced motion artefacts. With slice tracking [12,57], static tissue surrounding the heart (e.g. chest wall) may artificially turn into a moving structure, which leads to motion artefacts that affect image quality. With a 2D-T_2_-Prep, however, signal originating from such static background tissue is suppressed and slice tracking artefacts may be avoided. Similarly, self-navigation whole heart techniques [58,59] aggressively track motion over the entire range of respiratory excursions, which leads to an amplification of these artefacts. However, this may be avoided by combining self-navigation with a 2D-T_2_-Prep. Simultaneously, as a 2D-T_2_-Prep restricts the area from which signal is obtained to that of the heart, the accuracy of motion tracking may be improved as the self-navigation signal (i.e. the Fourier transform of an SI k-space profile) will not be contaminated by signal originating from the chest wall. Given that self-navigation techniques are frequently undersampled, they may thus be particularly well suited as a potential future application for the 2D-T2-Prep.

Another application that may particularly benefit from the 2D-T_2_-Prep includes the recently proposed Magnetic Resonance Fingerprinting, or MRF [60]. In MRF, a large number of highly undersampled, aliased images are rapidly acquired. By matching the signal evolution of these images to a predefined dictionary, a good quality parameter map can nonetheless be generated. However, as each image is highly undersampled, they also have the potential to be highly accelerated with parallel imaging. Combing MRF with parallel imaging reconstructions would reduce aliasing prior to the fitting routine, which may yield higher quality and/or more rapid dictionary fitting. Using a 2D-T_2_-Prep would then be particularly advantageous, as it would improve both the parallel imaging reconstruction (as demonstrated in this paper) and also reduce any additional aliasing from the undersampling. However, this interesting possibility remains to be tested.

Finally, it was also interesting to note that the peak improvement for the 2D-T_2_-Prep occurred at an acceleration factor of R = 4, as this corresponded to the degree of outer volume suppression (OVS) of the image. Specifically, a cylinder of radius 6.0 cm (area 113.1 cm^2^) within a 12.0 x 38.4 cm FoV (area 460.8 cm^2^), corresponds to an OVS area of approximately 75% in the accelerated direction (a *πr*^*2*^ circle vs. a 2r*FoV rectangle). In other words, the ratio of excited tissue area to suppressed tissue area, along the phase-encoding axis, is also approximately 4. This may suggest that for a given acceleration factor, one should suppress an equivalent portion of the image volume to achieve the maximal signal improvement. However, further validation is needed to unequivocally conclude this.

## Conclusion

A 2D-T_2_-Prep has been presented as a technique to improve GRAPPA-accelerated imaging of the heart, and it has been shown to significantly improve image quality in such acquisitions relative to the conventional T_2_-Prep. These results have been demonstrated in simulations, phantoms, and through *in vivo* MRA of the right coronary artery in healthy volunteers. The above technique should thus be considered for use in accelerated cardiac imaging, and perhaps explored for any form of accelerated image acquisitions where a local ROI is targeted and information from adjacent tissues is not required.

## Supporting Information

S1 File Data.xls Accompanying data for the manuscript, "A cylindrical, inner volume selecting 2D-T_2_-Prep improves GRAPPA-accelerated image quality in MRA of the right coronary artery". In Tab 1 ("Simulations"), the mean SNR of Blood, Fat, and Myocardium tissues may be seen, at various GRAPPA acceleration factors (corresponds to Fig 3). In Tab 2 ("Phantom VS %—PE"), one may see the percentage improvement in vessel sharpness vs acceleration factor using the 2D-T2-Prep, for a coronary model perpendicular to the accelerated direction (corresponds to Fig 5). In Tab 3 ("Phantom VS %—FE"), this is repeated for the same model, albeit it parallel to the accelerated direction (also corresponds to Fig 5). In Tab 4 ("Volunteers VS %"), on may see the vessel sharpness measurements made in the 10 volunteers studied in this manuscript, along with the corresponding statistical analysis.
(XLS)
